# Robot‐Assisted Radical Nephrectomy for Renal Cell Carcinoma in a Pelvic Kidney: Case Report

**DOI:** 10.1111/ases.70265

**Published:** 2026-02-22

**Authors:** Daiki Kawashima, Kojiro Ohba, Masaharu Oki, Tsuyoshi Matsuda, Kensuke Mitsunari, Tomohiro Matsuo, Ryoichi Imamura

**Affiliations:** ^1^ Department of Urology and Renal Transplantation Nagasaki University Hospital Nagasaki Japan; ^2^ Department of Urology National Hospital Organization Nagasaki Medical Center Nagasaki Japan

**Keywords:** pelvic kidney, radical nephrectomy, renal cell carcinoma, robot‐assisted

## Abstract

A 79‐year‐old Japanese man developed a low abdominal mass. Contrast‐enhanced computed tomography revealed a left‐sided pelvic kidney measuring 60 mm, suggestive of renal cell carcinoma. Abnormal vascular anatomy was also noted, including three renal arteries arising from the abdominal aorta, umbilical artery, and inferior mesenteric artery, respectively. On the basis of these findings, we performed robot‐assisted radical nephrectomy with placement of fluorescent ureteral stents to facilitate intraoperative identification of both ureters. The patient was positioned in steep Trendelenburg, and the pelvic kidney was removed safely. The console time was 156 min, and the estimated blood loss was 20 mL. To our knowledge, this is the first case of robot‐assisted radical nephrectomy for renal cell carcinoma arising in a pelvic kidney. Steep Trendelenburg positioning and placement of fluorescent ureteral stents were key to achieving a favorable surgical outcome.

## 1 Introduction

A pelvic kidney is a relatively rare congenital anomaly resulting from failed renal ascent during embryologic development [1, 2]. Renal cell carcinoma (RCC) arising in a pelvic kidney is particularly uncommon [3, 4, 5, 6]. To the best of our knowledge, there have been no previously published reports of robot‐assisted radical nephrectomy (RARN) for RCC in a pelvic kidney. Herein, we present the first known case of RARN for RCC in a pelvic kidney, highlighting the technical considerations and perioperative strategies that facilitated a favorable outcome.

## 2 Case Presentation

A 79‐year‐old Japanese man presented with lower abdominal pain and gross hematuria. His medical history included hypertension, benign prostatic hyperplasia, and cataracts, as well as prior appendectomy. There was no relevant family history. In September 2024, he consulted a local physician for hematuria and lower abdominal pain; a renal tumor was suspected, and he was referred to our hospital for further evaluation.

**FIGURE 1:**
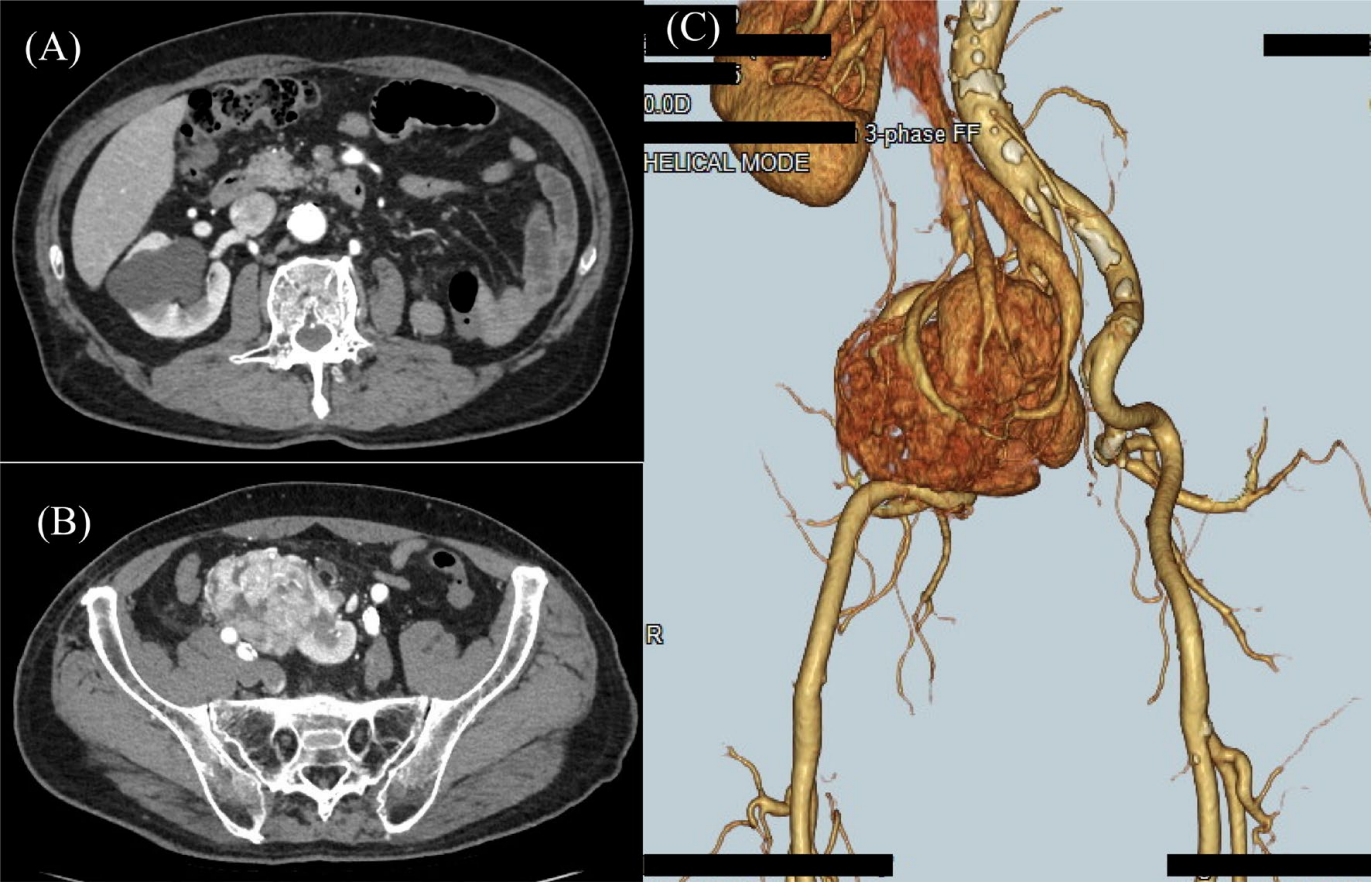
Preoperative CT findings of the pelvic kidney. (A) Axial contrast‐enhanced CT image shows the absence of the kidney in its normal anatomical position. (B) CT shows a 60 mm enhancing mass in the left pelvic kidney, which is located anterior to the sacrum. (C) Three‐dimensional CT shows clearly these findings. CT, computed tomography.

**FIGURE 2:**
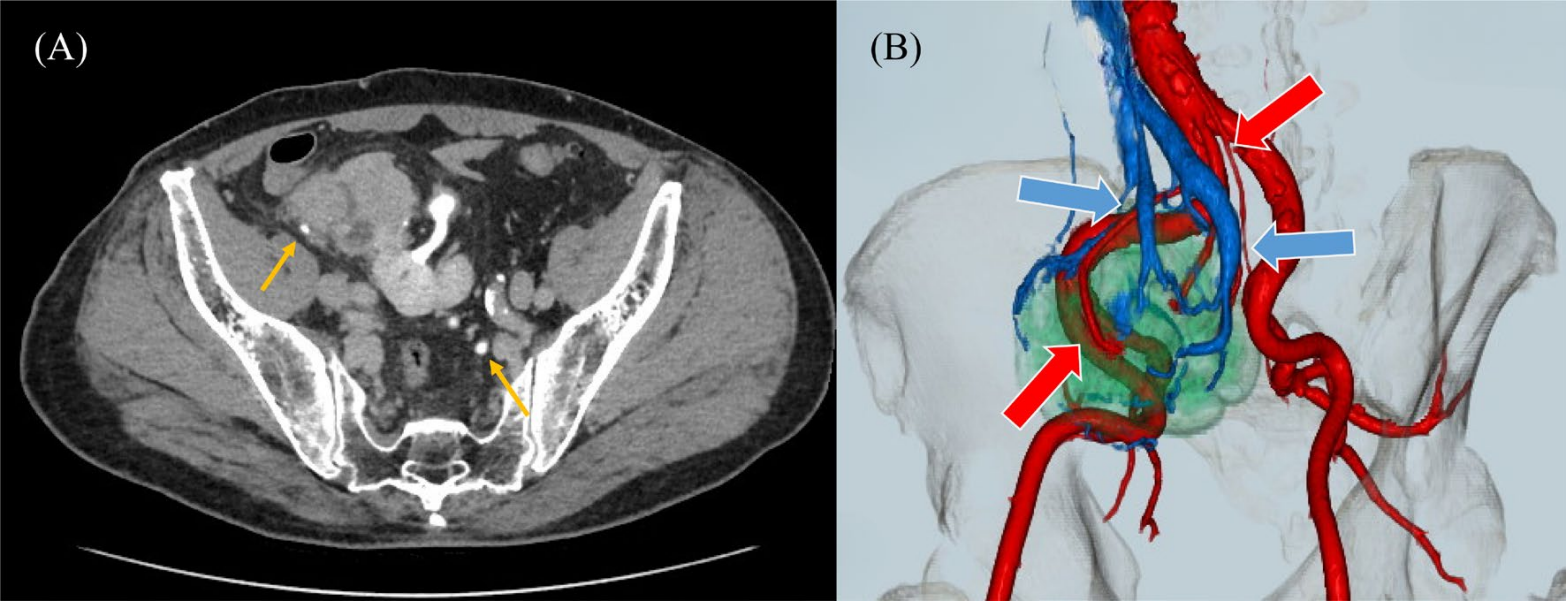
(A) The right ureter passes posterior to the left pelvic kidney, while the left ureter descends anteriorly (yellow arrows). (B) Three‐dimensional CT angiography showing anomalous renal vasculature. The left renal artery originates from the abdominal aorta and right common iliac artery (red arrows); multiple renal veins drain into the IVC (blue arrows). CT, computed tomography; IVC, inferior vena cava.

Contrast‐enhanced computed tomography revealed a left‐sided pelvic kidney measuring 60 mm and demonstrating early enhancement and delayed washout, which suggested RCC (Figure 1). The location of the ureter was close to the pelvic kidney, and abnormal vascular anatomy was also noted, including three renal arteries arising from the abdominal aorta, umbilical artery, and inferior mesenteric artery, respectively (Figure 2). On the basis of these findings, he was diagnosed with RCC of the left pelvic kidney, clinical stage T1bN0M0, and RARN was scheduled.

### 2.1 Surgical Findings

**FIGURE 3:**
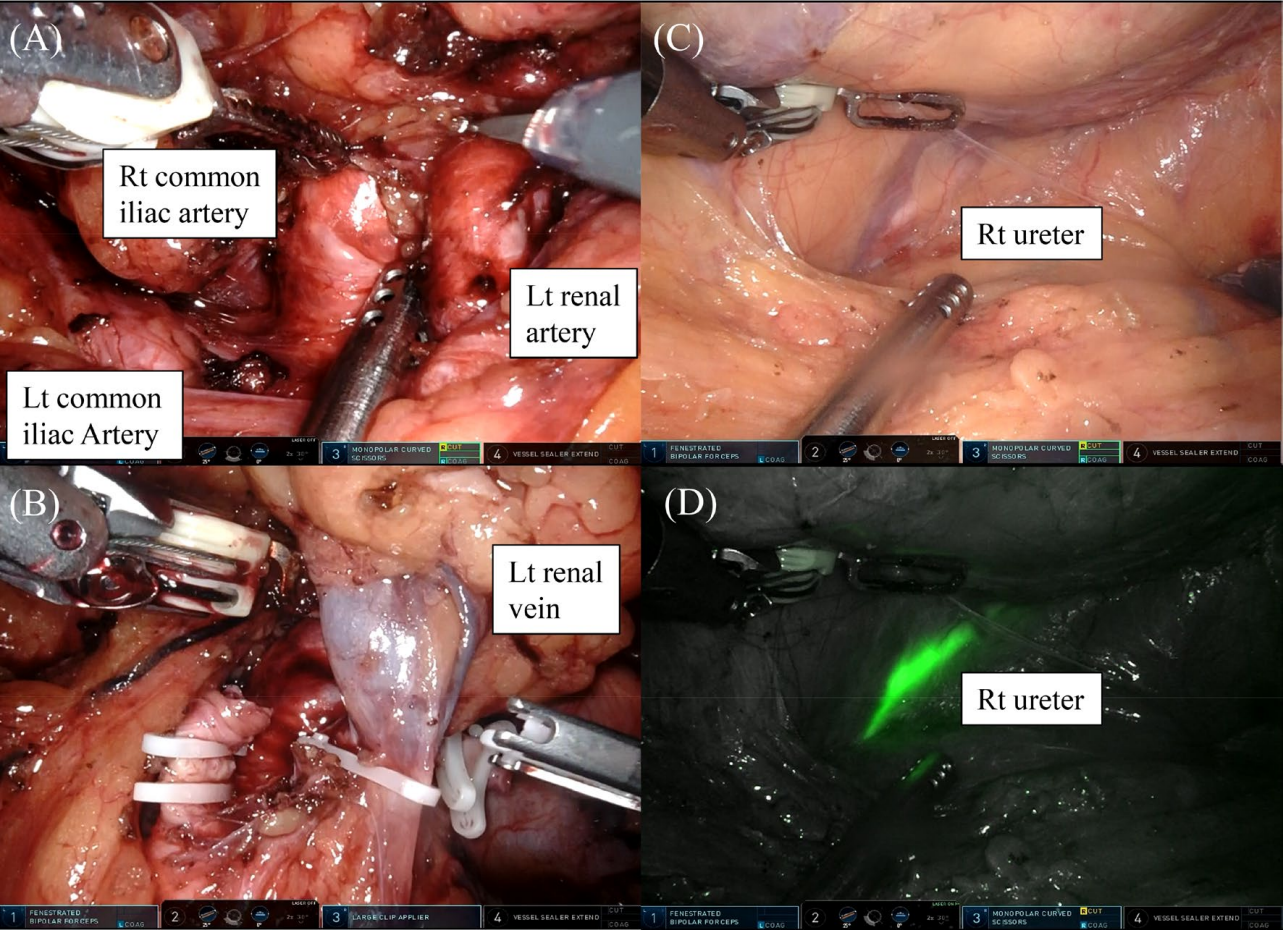
Intraoperative view during RARN. (A) The left renal artery was identified and dissected between the right and left common iliac arteries under robotic vision. (B) The Left renal vein was identified and ligated using clips. (C) The right ureter was observed, and (D) it was better visualized with fluorescent ureteral stent, which was detected with Firefly mode. RARN, robot‐assisted radical nephrectomy.

Prior to surgery, fluorescent ureteral stent catheters were placed to facilitate intraoperative identification of both ureters. The patient was positioned in steep Trendelenburg (25°), and five ports were used: four robotic ports of the da Vinci robotic system, with monopolar curved scissors in my right hand, fenestrated bipolar forceps in my left hand, and ProGrasp forceps as the 4th arm, and one assistant port. Dissection was initiated near the aortic bifurcation, where the left renal artery was identified and clipped (Figure 3). The pelvic kidney was then carefully mobilized from the sacrum, rectum, and right ureter, after which the left renal vein was identified and clipped (Figure 3). A prior appendectomy had little impact on the surgical procedure. The total operative time was 266 min, console time was 156 min, and estimated blood loss was 20 mL.

### 2.2 Histopathological Findings and Clinical Course After Surgery

Histopathological examination revealed that the tumor was a clear cell RCC (expansive type, 70 mm in maximum tumor diameter, Grade 2; WHO/ISUP grading system atypia classification, Grade 2; Fuhrman classification, LyX, VX, pT2a, INFa, eg, fc1, imX, rc‐inf0, rp‐inf0, s‐inf0). There was no perioperative complication. Without adjuvant therapy, 2 years after the operation, no recurrences have been identified.

## 3 Discussion

A pelvic kidney results from failed ascent during the 4th–8th weeks of gestation and is classified as a type of ectopic kidney [1, 7, 8]. Ectopic kidneys have a reported incidence of 0.08%–0.13%, with the pelvic kidney being the most common subtype [2, 4, 9]. This anomaly occurs more frequently on the left than on the right side and is slightly more prevalent in men than in women [1, 2].

Although many pelvic kidneys are asymptomatic, symptoms such as abdominal pain, hematuria, and urinary tract infections can appear, especially in the presence of tumors, stones, or obstruction [1, 2, 3]. Genitourinary anomalies are associated with pelvic kidneys in approximately 50% of cases, and other organ anomalies may coexist [7]. In our case, RCC was the only observed abnormality.

Surgical resection of RCC in a pelvic kidney poses distinct challenges because of aberrant vasculature and the confined pelvic anatomy. Renal arteries and veins may arise from the iliac vessels, aorta, or mesenteric branches, necessitating meticulous dissection and reliable vascular control [3, 4, 5, 6]. RARN offers important advantages in such anatomically complex cases. Compared with open or conventional laparoscopy, the robotic system provides enhanced dexterity, tremor filtration, and three‐dimensional, high‐definition visualization [1, 5]. Furthermore, the 4th arm was useful for making the optimal surgical view desired by the surgeon. These features facilitate precise dissection and vascular control in deep pelvic spaces.

Previously reported cases of RCC in ectopic pelvic kidneys are summarized in (Table 1) [1, 2, 5, 8, 10]. It is thought that partial nephrectomy could be performed with the advantage of robot‐assisted surgery, whereas radical nephrectomy may have been avoided due to the wide range of surgical view and the complex relationship with surrounding organs. In the present case, steep Trendelenburg positioning provided excellent pelvic exposure, a technique adapted from robotic pelvic oncologic procedures, such as radical prostatectomy [2]. Additionally, the fluorescent ureteral stent enabled real‐time identification of the ureter throughout the procedure, minimizing the risk of iatrogenic injury and aiding dissection near the rectum and contralateral ureter. Similar to our experience, Antonelli et al. demonstrated the feasibility of robotic techniques for complex pelvic renal anatomy, including robot‐assisted partial nephrectomy and bilateral pyelolithotomy in ectopic pelvic kidneys [1].

**TABLE 1:** Reported cases of renal cell carcinoma in ectopic pelvic kidneys.

Author (year)	Ectopic kidney type	Histology	Surgical position	Surgical approach	Notes
Karam et al. (2012)	Pelvic kidney	RCC (subtype NR)	Not specified	Robot‐assisted partial nephrectomy	Feasibility of robotic partial nephrectomy in ectopic pelvic kidney
Antonelli et al. (2019)	Bilateral pelvic kidneys; right renal mass	Clear cell RCC (pT1a)	Trendelenburg	Robot‐assisted partial nephrectomy with bilateral pyelolithotomy	Nephron‐sparing surgery in ectopic pelvic kidneys
Gharbi et al. (2019)	Ectopic pelvic kidney	Clear cell RCC (pT3a)	Spine	Open radical nephrectomy	Anomalous vascular network of ectopic kidney
Kumar et al. (2022)	Ectopic pelvic kidney	Clear cell RCC	Spine	Open radical nephrectomy	Complex RCC in ectopic pelvic kidney managed with radical nephrectomy
Badescu et al. (2025)	Sacral ectopic kidney	Chromophobe RCC (pT3a)	Spine	Open radical nephrectomy	Large chromophobe RCC in sacral ectopic kidney with literature review

Abbreviations: NR, not reported; pT1a, pathological type T1a; pT3a, pathological type T3a; RCC, renal cell carcinoma.

In conclusion, we described the first known case of RARN for RCC arising in a pelvic kidney. Steep Trendelenburg positioning, prior experience with robotic pelvic surgery, and placement of a fluorescent ureteral stent were keys to achieving a favorable surgical outcome. RARN represents a safe and effective option for managing RCC in pelvic kidneys.

## Author Contributions

All authors meet the ICMJE criteria for authorship. Daiki Kawashima, Kojiro Ohba, and Masaharu Oki conceived the study and wrote the manuscript. Tsuyoshi Matsuda collected the clinical data. Kensuke Mitsunari, Tomohiro Matsuo, and Ryoichi Imamura reviewed the literature. Kojiro Ohba performed the surgical procedure and critically revised the manuscript. All authors read and approved the final manuscript.

## Funding

The authors have nothing to report.

## Disclosure

All authors have approved the manuscript and agree with submission to this journal.

## Ethics Statement

This study was performed in accordance with the guidelines of the Declaration of Helsinki. Patient anonymity was maintained throughout the study.

## Consent

The authors have nothing to report.

## Conflicts of Interest

The authors declare no conflicts of interest.

## Data Availability

Data sharing is not applicable to this article as no datasets were generated or analyzed.

## References

[1] A. Antonelli , A. Veccia , C. Palumbo , et al., “Robot‐Assisted Partial Nephrectomy and Bilateral Pyelolithotomy in Ectopic Pelvic Kidneys,” Urology 128 (2019): 1–2, 10.1016/j.urology.2019.03.009. 30959118

[2] S. Kumar , P. Raj , A. Goyal , and D. Kriplani , “Multidisciplinary Management of a Complex Case of Renal Cell Carcinoma in Ectopic Pelvic Kidney,” Annals of the Royal College of Surgeons of England 104, no. 2 (2022): e57–e61, 10.1308/rcsann.2021.0139. 34813405 PMC10335231

[3] E. Yakut , D. Arslan , F. Akgun , et al., “A Rare Case of Paediatric Pelvic Ectopic Kidney Injury: Management,” Behcet Uz Cocuk Hastaliklari Dergisi 14, no. 1 (2024): 45–48, 10.4274/BMB.galenos.2023.2023-11-101.

[4] V. Fiaschetti , F. Lia , G. Claroni , G. Calabrese , and G. Simonetti , “Ectopic Pelvic Kidney: Evaluation With Multidetector CT Angiography,” Abdominal Imaging 39, no. 2 (2014): 215–221, 10.1007/s00261-013-0051-0.24173609

[5] A. R. Karam , M. P. Birkenbach , and A. K. Hemal , “Robot‐Assisted Partial Nephrectomy in an Ectopic Pelvic Kidney With Renal Cell Carcinoma,” Journal of Robotic Surgery 6, no. 2 (2012): 155–158, 10.1007/s11701-011-0295-1.27628279

[6] B. Akdogan , M. Ates , Y. Akin , et al., “Management of Renal Cell Carcinoma in Ectopic Kidneys: A Systematic Review,” Journal of Endourology 27, no. 8 (2013): 1035–1040, 10.1089/end.2012.0463.

[7] G. Bingham and M. G. Sulikowski , “Pelvic Kidney,” in StatPearls [Internet] (StatPearls Publishing, 2023), https://www.ncbi.nlm.nih.gov/books/NBK563239/. 33085386

[8] M. Gharbi , M. Chakroun , K. Chaker , et al., “Renal Cell Carcinoma in an Ectopic Pelvic Kidney: About a Case Report,” Urology Case Reports 23 (2019): 46–47, 10.1016/j.eucr.2018.12.006. 30581753 PMC6301969

[9] B. I. Chung and J. C. Liao , “Laparoscopic Radical Nephrectomy in a Pelvic Ectopic Kidney: Keys to Success,” JSLS 14, no. 1 (2010): 126–129. 20529537 10.4293/108680810X12674612765623 PMC3021314

[10] D. Badescu , O. C. Nechita , C. Toma , C. Sima , L. Nechita , and D. Nechita , “Chromophobe Renal Cell Carcinoma in Sacral Ectopic Kidney: Integrating Case Insights With a Review of Renal Ectopia Malignancies,” Annals of Clinical Medicine Case Reports 14, no. 11 (2025): 2344.

